# GCP III is not the “off-target” for urea-based PSMA ligands

**DOI:** 10.1007/s00259-023-06265-6

**Published:** 2023-05-16

**Authors:** Zhenghong Lee, Warren D. Heston, Xinning Wang, James P. Basilion

**Affiliations:** 1 Radiology, School of Medicine, Case Western Reserve University, Nuclear Medicine, Radiology, University Hospitals Cleveland Medical Center, 11100 Euclid Ave., Cleveland, OH 44106 USA; 2 Cancer Biology, Cleveland Clinic, Cleveland, OH 44195 USA; 3 Biomedical Engineering, Case Western Reserve University, Cleveland, OH 44106 USA

Dear Sir,

With great interest, we read a short communication in a recent issue of the *European Journal of Nuclear Medicine and Molecular Imaging* entitled *“Cross-reactivity to glutamate carboxypeptidase III causes undesired salivary gland and kidney uptake of PSMA-targeted small-molecule radionuclide therapeutics,”* by Lucaroni et al. [1]. In this communication, the authors postulate that the molecular interaction of Pluvicto™ ([^177^Lu]PSMA-617) with PSMA isozymes might represent the underlying cause of unwanted accumulation in healthy salivary glands and kidney. They based this on cross-reactivity of Glu-ureido-based inhibitors to proteins with high similarity to PSMA (i.e., isozymes, isoforms, and homologs) such as GCP III. There are, however, existing evidences that may not support their postulation.

Firstly, the inhibition constants of PSMA-targeted ligands (inhibitors) against GCP II (NAALAD or PSMA) and GCP III (NAALAD2) are well documented and much stronger for GCP II than GCP III [2]. For example, the selectivity of 2-PMPA against GCP II was tenfold higher than that against GCP III; DKFZ-PSMA-11 720-fold higher; and N-[[[(1*S*)-1-carboxy-3-methylbutyl]amino]carbonyl]-L-glutamic acid (ZJ-24) 160-fold higher [2]. The decrease of inhibitory potency of these PSMA ligands against GCP III may be explained in part by the replacement of Asn519 in GCP II with Ser509 in GCP III (metal sensitivity) and by the conformational change of three arginine residues, part of the “arginine patch,” in the S1 pocket of GCP III, compared to GCP II [3]. Given the 720-fold difference in its selectivity against GCP II vs. GCP III, it is doubtful for GCP III to be responsible for the very intense uptake of [^68^Ga]PSMA-11 in the salivary glands and kidneys.

Secondly, GCP III has a unique tissue distribution that is different from GCP II [4]. In addition to its expression in some female tissues, GCP III has high expression the human testis [5]. To date, the countless human clinical PSMA scans performed around the world have not produced a single image of detectible testicular uptake of the PMSA (GCP II)-targeted radioligands. Relevant to the Lucaroni et al. communication, mouse GCP III is predominantly expressed in the testis, heart, lung, and skeletal muscle, demonstrated by using cDNA, RNA, and enzymatic activity analysis [5, 6]. These organs, however, display little uptake of PSMA ligands during preclinical mouse scans. A note in parallel is that mouse PSMA is highly conserved with human PSMA with all the key amino acid residues involved in the PSMA binding pocket identical between the two species, and both exhibit similar substrate specificities [6]. However, in contrast to human PSMA, mouse PSMA is not expressed in mouse prostate [7], while PSMA expression in mouse salivary glands and kidneys is preserved at physiological expression levels.

Thirdly, it is suggested that internalization is necessary to achieve the large accumulation of PSMA radioligands into salivary and prostate cancer tissues. This internalization of PSMA is constitutive and seemed to be independent of small (short peptide) PSMA ligand binding or enzymatic activity [8] even though binding with monoclonal antibody would speed up the internalization by 3-fold [9]. The damage from Pluvicto™ to organs like the salivary glands is believed to be a consequence of retention of ^177^Lu through PSMA (GCP II) internalization with the bound radioligand [10]. However, internalization motifs have not been found for GCP III. Since GCP III has the smallest intracellular domain, the shortness and lack of conservation of internalization motifs with GCP III [10] suggest no internalization will occur for GCP III binding. Without internalization, the radionuclides (^177^Lu or ^225^Ac) would likely not be retained and concentrated to cause xerostomia (dry mouth) or other toxic side-effects. As a caveat for these studies, recombinant expression used for these studies to determine internalization is not the same as naturally occurring expression, and this has implications for internalization [11].

Given the important consequence of salivary gland uptake of PSMA-targeted radioligands for therapy and the discrepancies that exist in the literature summarized, we used a PSMA-knockout mouse model, in which GCP II expression is disabled [12]. These PSMA null mice were found to preserve some enzymatic activity of NAAG peptidase (from GCP III), for which the affinity of 2-PMPA was 88 nM in comparison to 1 nM for PSMA [12], which was in order with the above mentioned 2-PMPA binding results between GCP III and GCP II [2]. Our microPET imaging with [^68^Ga]PSMA-11 showed no uptake in the salivary glands and kidneys of the PSMA null mice compared to wild-type control mice (Fig. 1). The intact GCP III in the salivary glands and kidneys of these PSMA null mice did not seem to be able to account for the strong and sustained uptake of [^68^Ga]PSMA-11 seen in wild-type mice (Fig. 1). These results were in accordance with previous studies for kidney uptake in the same PSMA null mice using an iodinated PSMA ligand [13], which did not show kidney uptake although the salivary uptake (and xerostomia resulted from targeted radioligand therapy) was not an issue or focus at the time.

**Fig. 1:**
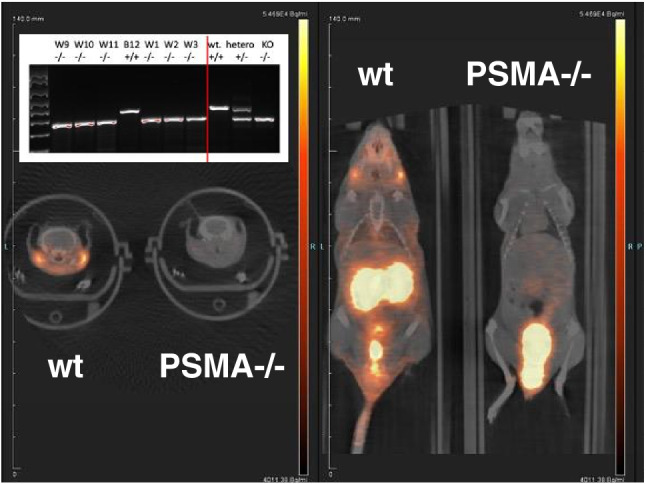
Comparison of [^68^Ga]PSMA-11 uptake between wild-type (wt) and PSMA null mice. PET/CT overlay of both axial (left) and coronal (right) cuts through the parotid (and some submandibular, axial view) glands. 200 $\mu$Ci (7.4 MBq) of the radioligand was injected intravenously via tail vein. A 5-min static microPET imaging was performed one-hour post-injection with the two mice scanned side-by-side and displayed at the same scale. The nasal-lacrimal uptake in wt mice (coronal view) was also noticed. *Upper left inset*: genotyping of several different mice to demonstrate knockout (KO), heterozygosity (hetero), or wild-type (wt) status. To the right of red vertical line are controls

So, how do we reconcile with the experimental results presented in the short communication [1]?

Staring from Fig. 1 of the Short Communication: is Compound 1 behaving exactly the same as PSMA-617? Is there any change in the lipophilicity (logP value) or charge of Compound 1 compared to the original PSMA-617? These would have implications in radiotracer kinetics and biodistribution and maybe internalization. As mentioned above, the binding affinity of 2-PMPA is tenfold better for GCP II (PSMA or NAALAD) than for GCP III (NAALAD2) [2]. The experimental results (K_D_) with Compound 1 were in line with similar fold difference (0.15 vs. 0.9) although the values of the constants were not the same, which might be explained by the use of recombinant expression discussed above.

In Fig. 2 (the antibodies): there are a few anti-PSMA antibodies (from Leica or Dako) used by many either clinically or experimentally, but why the authors used HPA010593, which did not detect any PSMA in the salivary glands according to Protein Atlas (https://www.proteinatlas.org/ENSG00000086205-FOLH1/tissue/salivary+gland)? Is HPA060802 specific for GCP III without cross-over to GCP II? Antibody specificity in differentiating GCP II vs. GCP II has been a problem [14].

Interestingly, a prior publication [15] listed an aminopeptidase, the NAALADase-like protein (NAALADaseL or NAALADL1), as one of the alternative target proteins for urea-based PSMA-targeting radioligands. Yet, this communication [1] showed in its Fig. 1C that NAALADaseL is not an “off-target” for the PSMA ligands.

## Data Availability

The PCR data and the reconstructed microPET images are available upon request.
